# Postoperative Axial Length Prediction Model in Children With Congenital Cataract and Intraocular Lens Implantation

**DOI:** 10.1155/joph/9948890

**Published:** 2025-01-28

**Authors:** Jialin Xu, Yunhui Yu, Yaqi Wang, Shenrong Zhang, Enze Liu, Wenjing Wang, Chenyuan Zhu, Jin Li

**Affiliations:** ^1^Department of Ophthalmology, Eye Hospital and School of Ophthalmology and Optometry, Wenzhou Medical University, Wenzhou, Zhejiang, China; ^2^National Clinical Research Center for Ocular Diseases, Wenzhou, Zhejiang, China; ^3^Department of Ophthalmology, Integrated Traditional Chinese and Western Medicine Hospital, Tianjin, China; ^4^Refractive Center, Eye Institute, Affiliated Hospital of Nantong University, 20 Xisi Road, Nantong, Jiangsu, China; ^5^Fittting Center, Qingdao Eye Hospital of Shandong First Medical University, Qingdao, Shandong, China

**Keywords:** axial length, congenital cataract/pediatric cataract, generalized estimating equation, intraocular lens implantation, prediction model

## Abstract

**Purpose:** To develop a prediction model for postoperative axial length (AL) in Asian children with congenital cataracts undergoing primary/secondary intraocular lens (IOL) implantation.

**Design:** Retrospective observational study.

**Methods:** Data were collected from children who underwent cataract surgery for congenital cataracts at the Eye Hospital of Wenzhou Medical University between 2006 and 2020. All participants completed preoperative and at least 1-year of postoperative follow-up. SPSS 26.0 software was used to analyze the variable factors affecting AL growth and the interactions among these factors. A generalized estimating equation (GEE) was employed to assess the correlation between the AL and related univariates over time. The univariate model was applied to build a multivariate model to predict the postoperative AL. Two validation sets were used to verify the accuracy of the formula.

**Results:** The study involved 86 children, accounting for 148 eyes. The median age at the time of surgery was 3.00 years, with a median age of 9.50 years at the final follow-up visit. The median duration of follow-up was 5.00 years. The preoperative and final follow-up mean ALs were 21.79 ± 1.77 and 23.36 ± 1.90 mm, respectively. Taking the predicted AL (*Y*) as the dependent variable and the age at surgery (*X*_1_), age at review (*X*_2_), and preoperative AL (*X*_3_) as the independent variables, the prediction model was established as *Y* = 0.20 − 0.473 × *X*_1_ + 0.446 × *X*_2_ + 0.993 × *X*_3_ − 0.014 × (*X*_2_ − *X*_1_)∗*X*_2_.

**Conclusions:** This model predicts AL growth in children following congenital cataract surgery and IOL implantation, helping ophthalmologists select appropriate IOL power.

## 1. Introduction

Congenital cataracts, present at birth or developing within the first year of life, are a leading cause of childhood blindness worldwide [1–3]. Intraocular lens (IOL) implantation effectively corrects refraction after congenital cataract surgery [4–7]. However, children's eyeballs continue to grow, and implanting an IOL with inappropriate power may hinder the development of visual function, often causing myopic drift [5, 8–10]. Therefore, it is extremely important to choose the most appropriate IOL power for the rapidly growing eyes.

To reduce postoperative myopia drift, ophthalmologists aim to retain appropriate hyperopia reserve based on the child's age as much as possible [11–13]. However, predicting axial length (AL) growth remains challenging as the eye continues to develop [14, 15]. Accurate AL prediction models are necessary to help ophthalmologists select the correct IOL power. McClatchey and Parks developed a logarithmic model that predicted final refraction within 3 diopters (*D*) for 24% of eyes in children under 2 years old and 77% for those over two, who underwent cataract surgery more than 20 years ago [16]. Trivedi et al. established an AL prediction model which involved 64 children (2–17.5 years old) with bilateral congenital cataract: postoperative AL = 1.93 + 0.91 × (baseline AL) − 0.07 × (baseline age) + 0.14 × (age at follow − up) − 0.005 × (baseline age) × (age at follow − up), which provided a basis for predicting the AL growth trend in children over 2 years old with primary IOL implantation [17]. Lottelli et al. also created an AL prediction model that involved both bilateral and unilateral cataract cases, introducing the concept of the rate of AL growth (RALG). RALG was defined as the slope calculated for each eye individually using linear regression based on at least two AL measurements at different ages, reflecting how much AL grows in relation to log_10_ corrected age. The model was expressed as follows: AL = preoperative eye axis + RALG × Log_10_ (follow-up age + 0.6)/(initial age + 0.6) [18]. However, these models were based on data from American and Brazilian children, and there were limited data applicable to Asian children. Our study included children who underwent cataract surgery with primary or secondary IOL implantation and a generalized estimating equation (GEE) analysis model to estimate the overall mean AL over time, aiming to develop a more accurate AL prediction formula applicable to Asian children.

The selection of IOL power mainly depends on children's age at surgery and preoperative AL, However, various factors can influence AL growth in children with congenital cataracts [19], complicating the selection of the appropriate IOL power. Therefore, this study collected and analyzed a large amount of data on the preoperative and postoperative AL of children with congenital cataracts. We developed a new AL prediction model using both single-factor and multifactor GEE. This model aims to assist pediatric surgeons in selecting the appropriate IOL power for Asian children.

## 2. Methods

### 2.1. Patients

We reviewed patients with congenital cataracts who underwent cataract surgery and IOL implantation at the Eye Hospital of Wenzhou Medical University from 2006 to 2020. We included children with a diagnosis of congenital cataracts who underwent cataract surgery with IOL implantation. All operations were performed by skilled cataract surgeons and all participants received monofocal IOLs. Children with cataracts due to congenital intrauterine infection, traumatic cataracts, developmental cataracts, metabolic cataracts, and other ocular diseases were excluded. None of the children in our study have a family history of high myopia or other genetic factors. We collected data on sex, age at surgery and last follow-up, surgical method, best corrected visual acuity (BCVA), preoperative and final AL, and intraocular pressure. This retrospective study was approved by the Ethics Committee of the Eye Hospital of Wenzhou Medical University and adhered to the Declaration of Helsinki.

### 2.2. AL Measurements

AL was measured using A-Mode Ultrasound Biometry (Quantel Medical Axis II PR Echograph, Japan) or IOLMaster 500 (Carl Zeiss Meditec AG, Jena, Germany) before surgery and at least 1 year after surgery. A-Mode Ultrasound or IOLMaster 500 measurements were taken by two well-trained technicians. Each eye was measured at least 10 times, and the average of these measurements was recorded.

### 2.3. Validation Sets

Two independent validation sets were used to verify the accuracy of the prediction model. Both validation sets adhered to the same inclusion and exclusion criteria applied to the study participants, and neither validation set was included in the original cohort of 148 eyes.

Validation set 1: 15 children (21 eyes) with congenital cataracts were randomly selected from the Eye Hospital of Wenzhou Medical University.

Validation set 2: 17 eyes of 11 children with congenital cataracts were randomly selected from the Hangzhou Branch of the same hospital.

Detailed demographic and clinical characteristics of both validation sets are provided in Supporting Tables 1 and 3.

### 2.4. Statistical Analysis

Continuous variables were tested for normality using the Kolmogorov–Smirnov test and expressed as mean ± standard deviation (SD), otherwise as median (quartile) or *n* (%). Comparative statistical analyses (including the Kolmogorov–Smirnov test, one-way ANOVA, Mann–Whitney *U* test, and unpaired *t*-test) were performed using SPSS software (Version 26.0; IBM Corporation). *p* values less than 0.05 were considered to be statistically significant. We used GEE to assess the correlations between future AL and each univariate variable over time. A univariate model was used to develop a multivariate model to predict the postoperative AL. The GEE is particularly valuable in ophthalmology, especially when considering the strong correlation between the two eyes of patients. This approach allows for the estimation of the overall mean AL over time while accounting for the interdependence of measurements from the same individual. Variables with a univariate *p* value of less than 0.01 were included in the multivariate GEE models to develop the final prediction model.

## 3. Results

### 3.1. General Information

A total of 86 children (148 eyes) who underwent cataract surgery and IOL implantation for congenital cataracts were included in the study. Of these, 72 (48.6%) had right eyes, 142 (95.9%) eyes underwent primary IOL implantation, and 65 (75.6%) patients had bilateral congenital cataracts. The median age at surgery was 3.00 (2.00, 6.00) years, the median age at the last follow-up was 9.50 (7.00, 12.00) years, and the median follow-up period was 5.00 (3.00, 8.00) years. The mean preoperative AL was 21.79 ± 1.77 mm, and the mean AL at the final follow-up was 23.36 ± 1.90 mm. The detailed characteristics of the patients are presented in Table 1.

### 3.2. AL Analysis by Age Group

The children were divided into four age groups: 0–2 years, > 2–4 years, > 4–6 years, and > 6 years. The AL of each group increased with age, with the longest final AL observed in the 4–6-years-old group (Table 2). There was no significant difference in the RALG among these groups (*p* = 0.104) (Table 3).

### 3.3. AL Comparison by Unilateral or Bilateral Cataracts

Patients were categorized into unilateral and bilateral congenital cataract groups. The unilateral congenital cataract group had a longer AL both preoperatively and at the last follow-up compared to the bilateral group (Table 4). There was no significant difference in the RALG between the two groups (Table 5).

### 3.4. Single-Factor and Multifactor GEE Analyses

The GEE was used to analyze the correlation between the AL and all univariates over time, including eye, sex, surgical method, age at surgery, age at review, follow-up years, BCVA, and preoperative AL. Statistically significant differences were found in age at surgery, age at review, and preoperative AL (*p* < 0.001) (Table 6). Based on univariate analysis and considering the pairwise interaction among the age at surgery, age at review, and follow-up years, the final results showed that the interaction between the age at review and follow-up years was statistically significant (Table 7).

### 3.5. AL Prediction Model

The final model predicted the coefficients (B values) for the final AL as follows: intercept (0.20), age at surgery (−0.473), age at review (0.446), preoperative AL (0.993), and interaction between age at review and follow-up years (−0.014). Using these variables, the predicted AL (*Y*) was calculated with age at surgery (*X*_1_), age at review (*X*_2_), and preoperative AL (*X*_3_) as independent variables. The model for predicting the AL is as follows:

(1) $$\begin{array}{c} Y=0.20-0.473\times X_{1}+0.446\times X_{2}+0.993\times X_{3}-0.014\times \left(X_{2}-X_{1}\right)*X_{2}. \end{array}$$

### 3.6. Performance of the Prediction Model in Validation Sets

Statistically significant differences were found in the age at the last follow-up (*p* < 0.001) between the groups by using a Mann–Whitney *U* test to compare the training set with Validation set 1 (Supporting Table 1). The age at surgery (*p* = 0.004) and age at the last follow-up (*p* < 0.001) were significantly different between the training set and Validation set 2 (Supporting Table 2).

Supporting Table 3 shows the general characteristics of the children in Validation set 1. Among the 15 children, 13 (61.9%) had right eyes, all 21 eyes underwent primary IOL implantation, and 6 (40.0%) had bilateral congenital cataracts. The median age at surgery was 4.00 (2.00, 6.50) years, the median age at the last follow-up was 5.00 (5.00, 9.00) years, and the median follow-up period was 3.00 (2.00, 3.00) years. The mean preoperative and final AL were 21.13 ± 0.92 and 22.02 ± 0.89 mm, respectively. For Validation set 1, a paired *t*-test comparing the observed final AL with the predicted AL from this study's model showed no statistically significant differences in children aged ≥ 2 years (*p* = 0.749) or across all ages (*p* = 0.332). Similarly, comparisons with the models by Trivedi et al. [17] (*p* = 0.137) and Lottelli et al. [18] (*p* = 0.754) revealed no significant differences (see Supporting Table 4).

Supporting Table 5 outlines the characteristics of the 11 children in the study. Among them, 7 (41.2%) had right eyes, 16 (94.1%) eyes underwent primary IOL implantation, and 6 (54.5%) had bilateral congenital cataracts. The median age at surgery was 2.00 (1.00, 3.50) years, the median age at the last follow-up was 4.00 (3.00, 4.50) years, and the median follow-up period was 1.00 (1.00, 2.00) years. The median preoperative AL was 20.65 (20.00, 22.71) mm, while the mean final AL was 21.89 ± 1.30 mm. For Validation set 2, the Wilcoxon test and paired *t*-test showed no significant differences between the observed and predicted AL for children aged ≥ 2 years (*p* = 0.536) and across all ages (*p* = 0.275), respectively. No significant difference was found when using the Wilcoxon signed–rank test to compare the actual AL with the predicted values of Trivedi et al.'s prediction model (*p* = 0.145). However, a comparison with the Lottelli et al. model using the paired *t*-test indicated a statistically significant difference (*p* = 0.038) (see Supporting Table 6).

## 4. Discussion

In this study, we analyzed the clinical data of 86 children with congenital cataracts who underwent cataract surgery with IOL implantation. First, univariate and multivariate GEE analyses showed significant differences in age at surgery, age at review, preoperative AL, and the interaction effects of age at review and the number of follow-up years. Therefore, we took the predicted AL (*Y*) as the dependent variable and the age at surgery (*X*_1_), age at review (*X*_2_), and preoperative AL (*X*_3_) as the independent variables and developed the following prediction formula: *Y* = 0.20 − 0.473 × *X*_1_ + 0.446 × *X*_2_ + 0.993 × *X*_3_ − 0.014 × (*X*_2_ − *X*_1_)∗*X*_2_. Next, children with congenital cataracts in Wenzhou Branch and Hangzhou Branch of Eye Hospital Affiliated to Wenzhou Medical University were randomly selected to form two validation sets. Statistical analysis showed no significant difference between the actual AL values at the last follow-up and those predicted by our model (*p* = 0.332 and *p* = 0.275). These results support the reliability of our model.

Previous studies also provided accurate AL prediction models for children after cataract surgery [17, 18]. Recently, Lottelli et al. conducted further validation of their model, confirming its high accuracy in predicting AL [20]. Trivedi's AL prediction model focused on children over 2 years old with bilateral congenital cataracts from the United States. Lottelli's model was developed based on 76 eyes of Brazilian children (aged 0–12 years) with unilateral or bilateral congenital or developmental cataracts, and it also included patients who had undergone piggyback IOL implantation. Our model is derived from the data on 148 eyes of Asian children with both unilateral and bilateral cataracts. These studies suggest that different models may have certain distinctions and may be applicable to different populations.

This prediction model represents the AL growth trend of children who have undergone surgery for congenital cataracts. The study by Trivedi et al. suggested that although sex and ethnicity were associated with the AL, no significant association was found in the final AL model [17]; therefore, we did not analyze these parameters. A study by Gordon and Donzis showed that the corneal curvature generally reached the adult range earlier than the AL, which usually stabilized at 6 months old [21]. The corneal curvature had a lower influence in the final model, and no significant difference was found. However, future studies could include initial corneal curvature data to improve prediction accuracy.

This was a retrospective observational study. Due to the limitation of the follow-up frequency and time, the follow-up interval for each child was different. In particular, 41 children were over 10 years old at the last follow-up, but only three were over 18 years old. The most ideal study method is to measure the AL of all children beginning preoperatively and continuing postoperatively at 3-month intervals until the AL stabilizes in adulthood. Moreover, a family history of AL development should be considered, as it may influence a child's AL growth.

Since children under 2 years old with congenital cataracts had poor cooperation, the A-mode ultrasound was used to measure their AL. Thus, the accuracy of the results cannot be guaranteed for younger children. Since we hoped to adapt our prediction formula to a wider population, patients under 2 years old were not excluded. Lenhart et al. proved that in adults, the choice of IOL power after A-mode ultrasound and IOLMaster measurement was not statistically significant [21]. Moreover, no study has proven the accuracy of the AL detection rate using the IOLMaster in young children; this requires further analysis and comparison.

Lambert et al. found no significant difference in the rate of refractive growth between patients with aphakic eyes and those with implanted IOLs after cataract surgery [15]. However, aphakic eyes with initially poorer visual acuity exhibited a higher rate of refractive growth, which may indicate that the AL growth and development in aphakic eyes is more obvious. McClatchey and Hofmeister showed that eyeball development (including the AL) begins in the embryonic period, rather than after birth [22]. Trivedi et al. also proved that the age of children under 18 months with congenital cataracts can be logarithmically transformed to enhance predictive accuracy [23]. However, since most of the children in our study were older than 2 years, logarithmic age conversion was not suitable. Our aim was to develop a prediction model suitable for all age groups; therefore, patients under 2 years old were included. The majority of children in our study underwent surgery at the age of 2, which aligns with the common practice of cataract surgeons performing surgery around this age. Consequently, our formula has wider suitability and higher accuracy for children aged 2 years and older. Nonetheless, we still need to include more children younger than 2 years to enhance the comprehensiveness of the model.

The eyeball of children develops rapidly, with the refractive state developing from +2.00 to +3.00 D at birth to around +1.00 D in adults. The trend of refractive state development toward myopia is termed myopia drift. Lv showed that younger children with congenital cataracts had a greater probability of postoperative myopia drift [24]. This is primarily due to the lens power decreasing during the normal growth and development of the eyeball, typically from 35.00 D to 18.00 D. Although the power of the IOL cannot be changed, the growth of the AL and the decrease in the corneal curvature result in great changes in the refractive state of the eyeball, especially in young children. When choosing IOLs for children with congenital cataracts, doctors reserve a certain degree of hyperopia for children, hoping to achieve binocular emmetropia in adulthood. Flitcroft et al. and Vasavada proposed that doctors should reserve the postoperative hyperopia power according to the patient's age, while this also had a risk of postoperative amblyopia [8, 25]. However, there was no consensus on how much hyperopia should be left, as postoperative amblyopia remains a risk. We compared the RALG among the groups at the age of surgery and found no significant differences. Moreover, the RALG was compared between unilateral and bilateral congenital cataract groups. As the age at surgery increased in the unilateral group, the AL growth rate decreased, which is consistent with the AL growth pattern. In the future, we should increase the sample size to keep the baseline age consistent and thus to confirm whether there is a difference in the RALG between the two groups.

Choosing the correct IOL power for children undergoing congenital cataract surgery remains challenging due to the unpredictable growth trend of AL. Our model incorporates the age at surgery, age at review, preoperative AL, and interaction of age at review and follow-up years. To simplify calculations, an application could be developed to input the children's preoperative AL, age at surgery, and predicted age. Incorporating new patient data into the application would further refine the model, increasing its accuracy.

Trivedi et al. proposed that in adults, IOL selection often requires consideration of both AL and corneal curvature to improve the accuracy of postoperative prediction [17]. However, while similar factors are considered for young children, the calculation model used for adults cannot be directly applied to children. Therefore, expanding the sample size in future studies would help to make the prediction model applicable to a broader population of children with congenital cataracts.

In this study, several limitations warrant consideration. First, the reliance on IOLMaster 500 and A-Mode Ultrasound Biometry for measuring AL in very young children may introduce some variability and potential inaccuracies, which could affect the reliability of our results. Second, the relatively small sample sizes for Validation sets 1 and 2 may raise questions about potential bias and the generalizability of our findings. Larger cohorts would enhance the robustness of our validation.

In conclusion, our model offers a reliable tool for predicting AL growth in children who undergo congenital cataract surgery. This can aid ophthalmologists in selecting appropriate IOL power, improving the likelihood of better visual outcomes for children postsurgery.

## Figures and Tables

**Table 1:** Patient characteristics.

Variable	Average/median/number (%)
Eye, right *n* (%)	72 (48.6%)
Gender, male *n* (%)	41 (47.7%)
Primary IOL implantation, *n* (%)	142 (95.9%)
Bilateral congenital cataract, *n* (%)	65 (43.9%)
Age at surgery, median (Q1, Q3) (year)	3.00 (2.00, 6.00)
Age at last follow-up, median (Q1, Q3) (year)	9.50 (7.00, 12.00)
Follow-up period, median (Q1, Q3) (year)	5.00 (3.00, 8.00)
Preoperative AL, mean ± SD (mm)	21.79 ± 1.77
Final AL, mean ± SD (mm)	23.36 ± 1.91
IOL type	
SN60WF, *n* (%)	29 (19.6%)
SN6AT, *n* (%)	15 (10.1%)
Softec HD, *n* (%)	16 (10.8%)
Other, *n* (%)	88 (59.6%)

*Note: Q*1, 25% percentile; *Q*3, 75% percentile.

Abbreviations: AL, axial length; IOL, intraocular lens; SD, standard deviation.

**Table 2:** Clinical data of different age groups.

Group	Number	Age at surgery (year)	Preoperative AL (mm)	Follow-up time after surgery (year)	Final AL (mm)
0–2	54	1.84 ± 0.36	21.00 ± 1.63	6.79 ± 3.11	23.02 ± 1.69
2–4	43	3.28 ± 0.56	21.87 ± 1.81	5.22 ± 3.32	23.41 ± 2.12
4–6	25	5.68 ± 0.48	22.48 ± 1.41	5.76 ± 2.76	23.82 ± 1.78
> 6	26	9.12 ± 2.18	22.65 ± 1.70	4.25 ± 2.83	23.50 ± 2.09

*Note:* Data are presented as mean ± standard deviation.

Abbreviation: AL, axial length.

**Table 3:** Comparison of RALG in different age groups.

Group	RALG	*F* value	*p* value
0–2	0.30 ± 0.16	2.089	0.104
2–4	0.35 ± 0.25		
4–6	0.25 ± 0.20		
> 6	0.25 ± 0.21		

*Note:* Data are presented as mean ± standard deviation; One-way ANOVA was performed in age groups.

Abbreviation: RALG, rate of axial length growth.

**Table 4:** Clinical data of unilateral and bilateral congenital cataract groups.

Group	Number	Eye number	Age at surgery (year)	Preoperative AL (mm)	Follow-up time after surgery (year)	Final AL (mm)
Unilateral	21	21	4.98 ± 2.67	22.96 ± 1.52	5.05 ± 2.89	24.25 ± 1.85
Bilateral	65	127	3 (2.6)	21.70 ± 1.75	9 (7.12)	23.30 ± 1.95
*p* value			0.071^a^	< 0.05^b^	0.845^a^	< 0.05^b^

*Note:* Data are presented as mean ± standard deviation or median (25% percentile and 75% percentile) based on the results of the normality test.

Abbreviation: AL, axial length.

^a^Mann–Whitney *U* test.

^b^Unpaired *t*-test.

**Table 5:** RALG of unilateral and bilateral congenital cataract groups.

Group	RALG	*p* value
Unilateral	0.29 ± 0.23	0.124
Bilateral	0.30 ± 0.20	

*Note:* Data are presented as mean ± standard deviation; Unpaired *t*-test was performed.

Abbreviation: RALG, rate of axial length growth.

**Table 6:** Single-factor GEE analysis.

Variable	*B*	Standard error	OR	95% CI of OR	*p* value
Eye (right vs. left)	−0.047	0.088	0.955	0.803∼1.135	0.598
Gender (male vs. female)	−0.101	1.916	0.904	0.621∼1.315	0.597
Surgical method (primary IOL implantation vs. secondary IOL implantation)	−0.172	0.473	0.842	0.333∼2.126	0.716
Age at surgery (year)	−0.297	0.050	0.743	0.674∼0.820	**< 0.001**
Age at review (year)	0.218	0.037	1.243	1.156∼1.337	**< 0.001**
Follow-up years (year)	0.124	0.064	1.132	0.998∼1.285	0.053
BCVA	−0.467	0.274	0.627	0.367∼1.073	0.088
Preoperative AL (mm)	0.994	0.050	2.702	2.448∼2.982	**< 0.001**

*Note:* The bold values indicate statistically significant differences found in age at surgery, age at review, and preoperative AL, as identified by the GEE analysis.

Abbreviations: AL, axial length; BCVA, best corrected visual acuity; CI, confidence interval; IOL, intraocular lens; OR, odds ratio.

**Table 7:** Multifactor GEE analysis.

Variable	*B*	95% CI of OR	*p* value
Intercept	0.200	0.136∼11.010	0.858
Age at surgery (year)	−0.473	0.521∼0.746	**< 0.001**
Age at review (year)	0.446	1.244∼1.960	**< 0.001**
Preoperative AL (mm)	0.993	2.469∼2.953	**< 0.001**
Follow-up years⁣^∗^ age at review	−0.014	0.972∼1.000	0.046

*Note:* The bold values indicate statistically significant differences found in age at surgery, age at review, and preoperative AL, based on univariate analysis.

Abbreviations: AL, axial length; CI, confidence interval; OR, odds ratio.

⁣^∗^Interaction.

## Data Availability

The datasets generated during and/or analyzed during the current study are available from the corresponding author on reasonable request.

## Supporting Information

Supporting Table 1. Comparison between the training set and Validation set 1.

Supporting Table 2. Comparison between the training set and Validation set 2.

Supporting Table 3. Patient characteristics in Validation set 1.

Supporting Table 4. Comparison between the final AL and predicted AL in Validation set 1.

Supporting Table 5. Patient characteristics in Validation set 2.

Supporting Table 6. Comparison between the final AL and predicted AL in Validation set 2.
