# Supplementary material for: Postoperative Axial Length Prediction Model in Children With Congenital Cataract and Intraocular Lens Implantation
Source: J Ophthalmol. 2025 Jan 28;2025:9948890. doi: 10.1155/joph/9948890 (PMC11824786; doi:10.1155/joph/9948890)
Supplement: Supporting Information — Additional supporting information can be found online in the Supporting Information section. [file 9948890.f1.docx]

**Supplementary Table 1.** Comparison between the training set and validation set 1

| **Data set** | **Age at surgery (year)** | **Age at last follow-up (year)** | **Preoperative AL (mm)** |
| --- | --- | --- | --- |
| Training set | 3.00(2.00,6.00) | 9.50(7.00,12.00) | 21.79±1.77 |
| Validation set 1 | 4.00(2.00,6.50) | 5.00(5.00,9.00) | 21.13±0.92 |
| *P* value | 0.419 | <0.001 | 0.089 |

AL=axial length; Mann-Whitney U test was performed.

**Supplementary Table 2.** Comparison between the training set and validation set 2

| **Data set** | **Age at surgery (year)** | **Age at last follow-up (year)** | **Preoperative AL (mm)** |
| --- | --- | --- | --- |
| Training set | 3.00(2.00,6.00) | 9.50(7.00,12.00) | 21.79±1.77 |
| Validation set 2 | 2.00(1.00,3.50) | 4.00(3.00,4.50) | 20.65(20.00,22.71) |
| *P* value | 0.004 | <0.001 | 0.129 |

AL=axial length; Mann-Whitney U test was performed.

**Supplementary Table 3.** Patient characteristics in validation set 1

| **Variable** | **Average/Median/Number（%）** |
| --- | --- |
| Eye, right, n (%) | 13(61.9%) |
| Gender, male, n (%) | 10(66.7%) |
| Primary IOL implantation, n (%) | 21(100.0%) |
| Bilateral congenital cataract, n (%) | 6(40.0%) |
| Age at surgery, Median (Q1, Q3) (year) | 4.00(2.00,6.50) |
| Age at last follow-up, Median (Q1, Q3) (year) | 5.00(5.00,9.00) |
| Follow-up years, Median (Q1, Q3) (year) | 3.00(2.00,3.00) |
| Preoperative AL, Mean ±SD (mm) | 21.13±0.92 |
| Final AL, Mean ±SD (mm) | 22.02±0.89 |
| IOL type |  |
| A1-UV, n (%) | 1(4.8%) |
| SN60WF, n (%) | 4(19.0%) |
| SN6CWS, n (%) | 9(42.9%) |
| SN6ATX, n (%) | 6(28.6%) |
| ZXR00, n (%) | 1(4.8%) |

AL=axial length; IOL=intraocular lens; Q1=25% percentile; Q3=75% percentile; SD= standard deviation

**Supplementary Table 4.** Comparison between the final AL and predicted AL in validation set 1

| **Study** | **Actual AL（mm）** | **Predicted AL（mm）** | **t/Z value** | ***P* value** |
| --- | --- | --- | --- | --- |
| This study  (≥2 years old)  This study  (All ages)  Lottelli’s study | 22.01±0.94  22.02±0.89  22.02±0.89 | 21.88±0.87  21.89±0.85  22.07±0.79 | 0.439  0.994  -0.318 | 0.749  0.332  0.754 |
| Trivedi’s study | 22.01±0.94 | 21.58±0.87 | 0.774 | 0.137 |

AL= axial length; Data are presented as mean ±standard deviation; A paired test was performed.

**Supplementary Table 5.** Patient characteristics in validation set 2

| **Variable** | **Average/Median/Number（%）** |
| --- | --- |
| Eye, right, n (%) | 7 (41.2%) |
| Gender, male, n (%) | 5(45.5%) |
| Primary IOL implantation, n (%) | 16(94.1%) |
| Bilateral congenital cataract, n (%) | 6(54.5%) |
| Age at surgery, Median (Q1, Q3) (year) | 2.00(1.00,3.50) |
| Age at last follow-up, Median (Q1, Q3) (year) | 4.00(3.00,4.50) |
| Follow-up period, Median (Q1, Q3) (year) | 1.00(1.00,2.00) |
| Preoperative AL, Median (Q1, Q3) (mm) | 20.65(20.00,22.71) |
| Final AL, mean ±SD (mm)  IOL type  A1-UV, n (%)  AMOAR40e, n (%)  ZCB00, n (%) | 21.89±1.30  10(58.8%)  6(35.3%)  1(5.9%) |

AL=axial length; IOL=intraocular lens; Q1=25% percentile; Q3=75% percentile; SD= standard deviation

**Supplementary Table 6.** Comparison between the final AL and predicted AL in validation set 2

| **Study** | **Actual AL（mm）** | **Predicted AL（mm）** | **t/Z value** | ***P* value** |
| --- | --- | --- | --- | --- |
| This study  (≥2 years old)  This study  (All ages)  Lottelli’s study  Trivedi’s study | 23.15(21.36,23.76)  21.89±1.30  21.89±1.30  23.15(21.36,23.76) | 22.63±1.20  21.77±1.32  22.17±1.22  22.07±1.13 | -0.618  1.131  -2.260  -1.4 | 0.536  0.275  **0.038**  0.145 |

AL= axial length; Data are presented as mean ±standard deviation or median (25% percentile, 75% percentile); The Wilcoxon signed-rank test and a paired t-test were performed.
